# Prospects of Chimeric Antigen Receptor T-Cell Therapy in Myelofibrosis: From Immunopathogenesis to Therapeutic Strategies

**DOI:** 10.3390/cancers18091493

**Published:** 2026-05-06

**Authors:** Lulu Kong, Chunling Fu, Lianggui Song, Wenxiao Wang, Mengchu Ji, Fei Li, Xiaofeng Shi, Wei Chen

**Affiliations:** 1 The Affiliated Hospital of Xuzhou Medical University, Department of Haematology, Institute of Blood Diseases, Key Laboratory of Bone-Marrow Stem Cells, Xuzhou 221000, China; 304102110092@stu.xzhmu.edu.cn (L.K.); fucl@xzhmu.edu.cn (C.F.); 304102110102@stu.xzhmu.edu.cn (L.S.); 302510210093@stu.xzhmu.edu.cn (W.W.); 303102110075@stu.xzhmu.edu.cn (M.J.); 2 Clinical Medicine Research Center, Jiangsu Province (Suqian) Hospital, Suqian 223800, China; zhaoyy@yctu.edu.cn; 3 The Second Affiliated Hospital of Nanjing Medical University, The Second Clinical Medical School of Nanjing Medical University, Nanjing 210011, China

**Keywords:** myelofibrosis, chimeric antigen receptor T (CAR-T)-cell therapy, immune microenvironment remodeling, clonal hematopoiesis, fibrotic bone marrow niche

## Abstract

Myelofibrosis (MF) is a myeloproliferative neoplasm characterized by clonal hematopoietic dysregulation, amplification of chronic inflammation, and progressive remodeling of the bone marrow fibrotic niche, clinically manifesting as bone marrow failure, splenomegaly, and systemic inflammatory symptoms. Although Janus kinase (JAK) inhibitors can alleviate symptom burden and reduce spleen size, they have limited capacity to eradicate malignant clones or reverse fibrosis. Allogeneic hematopoietic stem cell transplantation remains the only potentially curative option; however, its application is constrained by advanced age, comorbidities, unavailable donor, and transplant-related risks. Therefore, the development of disease-modifying therapeutic strategies has become a central focus in MF research. Chimeric antigen receptor T (CAR-T)-cell therapy has demonstrated robust efficacy across various hematologic malignancies. Its application in MF holds the potential not only to selectively eliminate malignant hematopoietic clones but also to modulate the immunosuppressive and profibrotic microenvironment through advanced cellular engineering, thereby enabling a dual therapeutic paradigm involving both clonal control and microenvironmental reprogramming. In this context, potential targets and pathways include CD123, myeloproliferative leukemia protein (MPL), fibroblast activation protein (FAP), the TGF-β signaling axis, the CXCR4–CXCL12 niche-regulatory axis, and molecules associated with myeloid-derived suppressor cells (MDSCs) and tumor-associated macrophages (TAMs). Future strategies may optimize both efficacy and safety through combinatorial approaches, including integration with JAK inhibitors, development of armored CAR-T constructs, and bridging to hematopoietic stem cell transplantation. Collectively, CAR-T-cell therapy offers a promising avenue for shifting MF management from symptomatic control toward true disease modification.

## Simple Summary

Myelofibrosis is a chronic myeloid malignancy characterized by abnormal clonal hematopoiesis, persistent inflammation, and progressive fibrosis of the bone marrow. Current treatments, particularly Janus kinase inhibitors, can improve splenomegaly and disease-related symptoms but generally have limited ability to eliminate malignant clones or reverse the fibrotic microenvironment. Allogeneic hematopoietic stem cell transplantation remains the only potentially curative option, but its use is restricted by patient age, comorbidities, donor availability, and transplant-related risks. Chimeric antigen receptor T-cell therapy has transformed the treatment of several hematologic malignancies and may provide a new disease-modifying strategy for myelofibrosis. In this review, we discuss the immunopathological basis of myelofibrosis, potential CAR-T targets involving malignant hematopoietic clones and the fibrotic or immunosuppressive bone marrow niche, and major translational challenges such as on-target, off-tumor toxicity and impaired CAR-T persistence. We also summarize future strategies, including armored or controllable CAR-T designs, combination with JAK inhibitors, and use as a bridge to transplantation.

## 1. Overview of Myelofibrosis

Myelofibrosis (MF), encompassing primary myelofibrosis (PMF) and secondary myelofibrosis (SMF) evolving from polycythemia vera (PV) or essential thrombocythemia (ET), is a clonal myeloproliferative neoplasm (MPN) [1,2]. Although MF is a relatively rare hematologic malignancy, its incidence increases with age. Clinically, MF is characterized by progressive bone marrow fibrosis, extramedullary hematopoiesis, peripheral cytopenias, splenomegaly, and a substantial systemic symptom burden, all of which significantly impair quality of life [3,4]. The clinical course of MF is highly heterogeneous; based on clinical and molecular risk stratification models, the median overall survival (OS) ranges from approximately 3 to 7 years. A subset of patients may progress to acute myeloid leukemia (AML), particularly those in higher-risk categories, in whom leukemic transformation represents a major cause of mortality [5,6,7].

Current therapeutic options for MF remain limited. Janus kinase inhibitors (JAKis) have become the standard of care for symptom control and spleen volume reduction; however, they do not effectively eradicate the malignant hematopoietic clone, reverse BMF, or prevent leukemic transformation [1,2,8]. Moreover, long-term treatment is frequently complicated by resistance or intolerance. Beyond first-generation JAK2i, momelotinib (JAK1/2 inhibitor) has demonstrated clinical benefit in the phase III MOMENTUM trial, particularly in addressing splenomegaly and anemia [9,10]. Pacritinib (JAK2/FLT3 inhibitor) has shown efficacy in patients with severe thrombocytopenia [11]. In the context of “JAK inhibition plus disease-modifying strategies,” the combination of the BET inhibitor pelabresib with ruxolitinib achieved higher spleen response rates and signals of bone marrow morphological improvement in the phase III MANIFEST-2 trial [12]. Similarly, navitoclax (BCL-2 inhibitor) in combination with ruxolitinib demonstrated improvements in spleen size and BMF in the REFINE study [13,14]. In addition, anemia-targeting agents such as luspatercept have shown supportive clinical activity [15]. Allogeneic hematopoietic stem cell transplantation (allo-HCT) remains the only potentially curative treatment; however, its applicability is limited by advanced patient age, ECOG (Eastern Cooperative Oncology Group Performance Status) score, comorbidities, donor availability, and substantial transplant-related morbidity and mortality [4,16].

Overall, existing therapeutic strategies are largely focused on symptom control and signaling pathway inhibition, with limited capacity to simultaneously address clonal expansion and immune–stromal disequilibrium. In this context, the introduction of cellular immunotherapies such as chimeric antigen receptor T (CAR-T) cells into MF represents a rational and promising approach. CAR-T therapy not only provides the potential for selective targeting of pathogenic clones but may also enable multidimensional modulation of MF biology through enhancement of effector immunity, alleviation of immunosuppression, and remodeling of the fibrotic microenvironment.

## 2. Advances of CAR-T Therapy in Hematologic Malignancies

In recent years, CAR-T-cell therapy has achieved transformative progress in hematologic malignancies. At the preclinical level, continuous structural optimization and cellular engineering have enhanced antitumor efficacy [17,18]. A typical CAR construct consists of an extracellular antigen-recognition domain (most commonly a single-chain variable fragment, scFv), a hinge/transmembrane region, and intracellular signaling modules. The CD3ζ chain provides the primary activation signal, whereas costimulatory domains such as CD28 or 4-1BB deliver signal 2 and shape downstream signaling programs [19]. Specifically, CD28-based CARs favor rapid expansion and effector differentiation via activation of the PI3K–AKT–mTOR axis, whereas 4-1BB-based CARs promote mitochondrial fitness and persistence through TRAF–NF-κB-associated pathways. These differences in costimulatory domains critically influence killing activity, T-cell exhaustion, memory formation, and long-term disease control [20,21,22]. Although CAR-T-cell design has progressed from first-generation constructs to more advanced second-, third-, and fourth-generation platforms, later generations are not necessarily superior in all contexts. The optimal CAR architecture depends on the target antigen, antigen density, normal tissue expression, tumor lineage, disease burden, and microenvironmental constraints [18]. Second-generation CARs containing CD28 or 4-1BB costimulatory domains remain the most clinically validated platforms. CD28-based CARs generally favor rapid expansion and effector differentiation, whereas 4-1BB-based CARs promote persistence, mitochondrial fitness, and memory-like features [19,20]. Third-generation CARs may increase signaling intensity, whereas fourth-generation or armored CAR-T cells may be useful in immunosuppressive niches, but their superiority requires context-specific validation [23]. Therefore, in MF, each candidate target should be individually evaluated to identify the most appropriate CAR design. This includes optimization of scFv affinity and specificity, hinge and transmembrane regions, costimulatory domains, CD3ζ signaling strength, activation threshold, persistence, cytotoxicity, exhaustion profile, and off-tumor toxicity. Such antigen-specific investigation is particularly important because MF-associated targets often overlap with normal hematopoietic or stromal cells.

Clinically, CAR-T therapy has demonstrated robust efficacy in B-cell acute lymphoblastic leukemia (B-ALL), diffuse large B-cell lymphoma (DLBCL), and multiple myeloma (MM), and is progressively being incorporated earlier in treatment paradigms rather than being restricted to relapsed/refractory (R/R) settings [24,25,26,27,28]. Studies from multiple centers, including the Affiliated Hospital of Xuzhou Medical University, have shown that humanized CD19-directed CAR-T cells can achieve high complete remission (CR) rates in R/R B-ALL [29]. Similarly, B-cell maturation antigen (BCMA)-targeted CAR-T therapy has significantly improved overall response rates (ORR) and progression-free survival (PFS) in patients with relapsed/refractory multiple myeloma (RRMM). Importantly, CAR-T-associated toxicities, including cytokine release syndrome (CRS) and immune effector cell-associated neurotoxicity syndrome, are generally manageable with optimized lymphodepletion regimens and dose modulation. Collectively, CAR-T therapy is evolving from a single-target cytotoxic modality toward a more integrated immunotherapeutic platform incorporating immune microenvironment modulation [18,30,31,32]. Several CAR-T-cell products targeting CD19 or BCMA have been approved for B-cell malignancies and multiple myeloma, demonstrating the clinical feasibility and therapeutic impact of this platform [24,28]. However, CAR-T-cell therapy for MF remains at an exploratory stage, and its successful translation will require MF-specific target selection, safety optimization, and microenvironment-directed engineering strategies [33].

**Figure 1:**
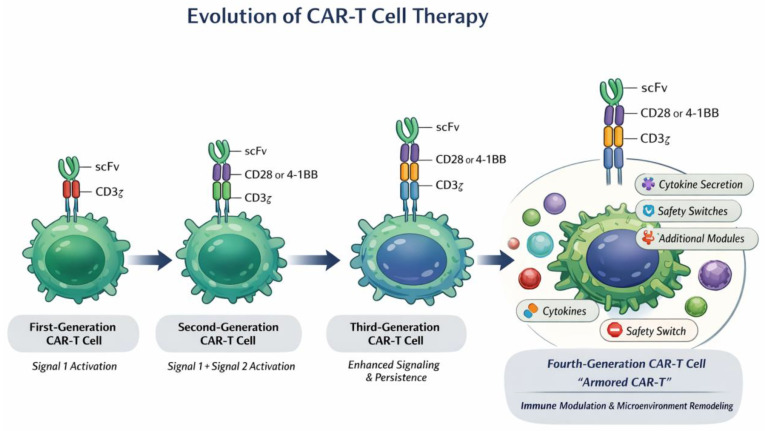
Evolution of CAR-T-cell design and functional optimization.

In the context of MF, a central challenge lies in the identification of suitable therapeutic targets. Ideally, such targets should be highly expressed on malignant clones or key pathogenic cell populations while sparing normal hematopoietic stem and progenitor cells (HSPCs) to minimize hematologic toxicity. However, MF-associated targets, including CD123 and MPL, are also expressed on normal hematopoietic cells, posing a dual challenge of specificity and safety [34,35,36]. Furthermore, the fibrotic bone marrow characteristic of MF represents a major barrier to effective immunotherapy. Dense extracellular matrix deposition, infiltration of immunosuppressive cell populations, and elevated levels of profibrotic cytokines collectively impair T-cell trafficking and effector function. Accordingly, current research is shifting from solely targeting malignant cells toward comprehensive microenvironmental reprogramming. Armored CAR-T cells, engineered to locally secrete cytokines or deliver additional immunomodulatory signal 3, have shown promise in enhancing T-cell infiltration and overcoming immunosuppression [23,37]. In parallel, strategies targeting non-malignant components of the MF microenvironment—such as cancer-associated fibroblasts (CAFs) or immunosuppressive myeloid populations (e.g., MDSCs and tumor-associated macrophages, TAMs)—are being actively explored [38,39]. By disrupting stromal and immunosuppressive networks, these approaches may reduce therapeutic resistance and improve the durability of combination immunotherapies. Taken together, these advances highlight the progressive evolution of CAR-T-cell design from basic cytotoxic constructs toward multifunctional engineered platforms capable of modulating both tumor cells and the surrounding microenvironment. The structural and functional iterations of CAR-T cells are schematically illustrated in Figure 1.

Building on these advances, the following sections first outline the immunopathological features of MF that create both opportunities and barriers for CAR-T-cell therapy, and then discuss candidate targets, translational challenges, and future engineering strategies.

## 3. Immunopathological Basis of Myelofibrosis

### 3.1. Clonal Hematopoietic Abnormalities

Clonal hematopoiesis represents a fundamental molecular and pathological basis of MF. Accumulating evidence indicates that driver mutations in JAK2, CALR, and MPL constitutively activate the Janus kinase–signal transducer and activator of transcription (JAK-STAT) signaling pathway, thereby conferring proliferative and survival advantages to HSPCs and promoting the emergence of self-renewing malignant clones. Among these, the JAK2 V617F mutation (a valine-to-phenylalanine substitution at codon 617 of Janus kinase 2) enhances the production of proinflammatory cytokines and chemokine signaling, whereas CALR mutations aberrantly activate MPL through altered ligand–receptor interactions, collectively driving disease progression [40,41].

Concomitantly, malignant clones actively remodel the bone marrow microenvironment through the secretion of profibrotic and proinflammatory mediators, including transforming growth factor-β (TGF-β), platelet-derived growth factor (PDGF), and interleukin-1β (IL-1β). These factors induce fibroblast activation, reticulin and collagen deposition, angiogenesis, and the expansion of immunosuppressive cell populations [42,43,44]. As a result, the bone marrow niche transitions from a hematopoiesis-supportive state to a fibrotic and immunosuppressive milieu, which is widely recognized as a key pathological mechanism underlying the progression from chronic clonal hematopoiesis to bone marrow failure and extramedullary hematopoiesis in MF [45].

### 3.2. Immune Microenvironment Dysregulation

In PMF, persistent inflammation and tumor-associated myeloid expansion synergistically drive immune microenvironmental imbalance [46]. T cells exhibit a phenotype characterized by enhanced activation yet functional exhaustion, with upregulation of programmed cell death protein 1 (PD-1) observed in both peripheral blood and bone marrow T cells, correlating with disease burden and clinical outcomes. Recent studies have further demonstrated co-expression of multiple inhibitory receptors—including PD-1, T-cell immunoglobulin and mucin-domain containing-3 (TIM-3), lymphocyte activation gene-3 (LAG-3), and T-cell immunoreceptor with Ig and ITIM domains (TIGIT)—highlighting the central role of immune checkpoint pathways in establishing an immunosuppressive network [47,48].

In JAK2 V617F -driven MF, natural killer (NK) cells display impaired maturation and reduced cytotoxic function, thereby weakening immune surveillance against malignant clones. Single-cell transcriptomic analyses have also revealed dysregulated NK cell-associated activation and exhaustion signatures, which may be further modulated by therapeutic exposure [44]. In parallel, expansion of immunosuppressive myeloid populations and regulatory lymphocyte subsets reinforces tumor immune evasion. Increased levels of polymorphonuclear myeloid-derived suppressor cells (PMN-MDSCs) have been identified in both peripheral blood and the spleen of patients with PMF, correlating with disease progression [49].

Moreover, mutant CALR has been shown to activate the TGF-β signaling axis, promoting the expansion of regulatory T cells (Tregs) within the bone marrow microenvironment [50]. This results in a dual-layered immunosuppressive network characterized by MDSC-mediated myeloid suppression and Treg-mediated adaptive immune regulation, ultimately impairing antitumor immune surveillance and facilitating sustained clonal evolution [51].

### 3.3. Cytokine Storm-like Inflammatory Milieu

In PMF and related MPNs, disease progression occurs within a cytokine milieu resembling a chronic “cytokine storm-like” state. Key inflammatory mediators, including interleukin-6 (IL-6), tumor necrosis factor-α (TNF-α), and TGF-β, form a central axis driving both fibrosis and clonal expansion [41,52]. IL-6, a prototypical proinflammatory cytokine, activates the JAK/STAT3 pathway, enhancing myeloid cell proliferation and amplifying inflammatory cascades [53,54]. Elevated IL-6 levels are strongly associated with systemic inflammatory symptoms and adverse clinical outcomes. Transcriptomic and single-cell sequencing analyses have further demonstrated enrichment of IL-6-related signaling pathways in the PMF bone marrow microenvironment, correlating positively with the degree of fibrosis [40,44].

**Figure 2:**
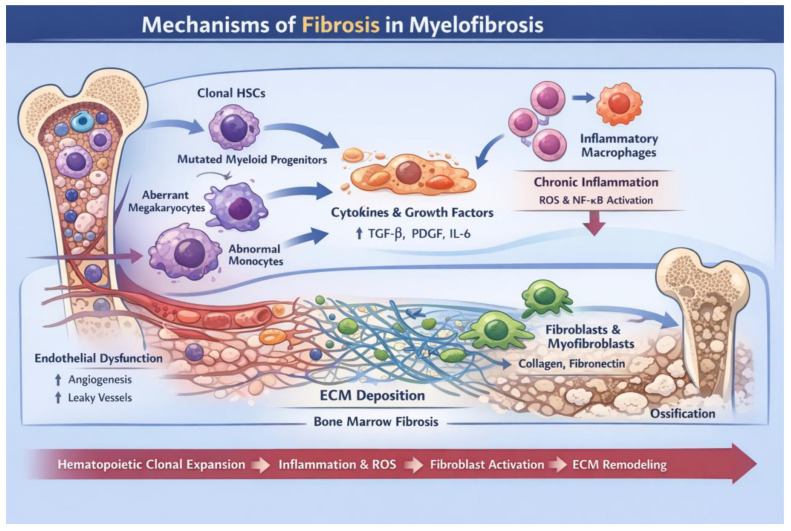
Mechanisms of fibrosis in myelofibrosis.

TNF-α, persistently elevated in MF, sustains chronic inflammation through activation of the nuclear factor-κB (NF-κB) pathway, while suppressing normal HSPCs and relatively favoring the expansion of mutant clones [55,56]. TGF-β is widely recognized as a key effector molecule in bone marrow fibrosis, primarily derived from abnormal megakaryocytes and activated myeloid cells. Through SMAD-dependent signaling pathways, TGF-β induces fibroblast activation, extracellular matrix deposition, and collagen synthesis, thereby promoting stromal remodeling [57]. In addition, mutant CALR further enhances TGF-β signaling, reinforcing immunosuppression and structural reorganization of the bone marrow niche. Collectively, these interconnected mechanisms establish a proinflammatory, immunosuppressive, and profibrotic microenvironment that underpins disease progression and therapeutic resistance in MF [50]. As shown in Figure 2, MF progression can be conceptualized as a stepwise process involving clonal expansion, cytokine-driven inflammation, fibroblast activation, and extracellular matrix remodeling, ultimately resulting in bone marrow fibrosis and osteosclerosis.

## 4. Potential Targets of CAR-T Therapy in Myelofibrosis

### 4.1. Targeting Malignant Hematopoietic Clones

In MF, the application of CAR-T-cell therapy for direct eradication of malignant hematopoietic clones remains at an early exploratory stage. A major challenge lies in the substantial overlap of antigen expression between candidate targets and HSPCs. CD34, a canonical marker of HSPCs, exhibits highly overlapping expression in both normal and malignant compartments, lacking sufficient differential antigen density to enable selective targeting. Consequently, CD34-directed strategies pose a significant risk of hematopoietic stem cell depletion and long-term bone marrow failure, limiting their translational feasibility. Arruda et al. developed a CD34/CD3 bispecific T-cell engager (BiTE), demonstrating CD34-dependent T-cell activation and potent cytotoxicity against leukemic cells in vitro [58]. This approach effectively eliminated CD34^+^ blasts derived from patients with AML and significantly reduced leukemic burden in the bone marrow and spleen in NSG-SGM3 xenograft models [59]. These findings support the feasibility of targeting the CD34 axis as an immunotherapeutic strategy against leukemic stem cells (LSCs) in AML and myelodysplastic syndromes (MDSs). In MF, which similarly originates from aberrant HSPCs, elevated circulating CD34^+^ cells represent a hallmark biological feature. Notably, circulating CD34^+^ cells from patients with PMF have been shown to induce myeloid-biased hematopoiesis and bone marrow fibrosis in immunodeficient mouse models, underscoring their critical role in disease development and maintenance [60,61]. Therefore, the preclinical success of CD34-directed T-cell redirection in AML/MDS provides a translational rationale for targeting pathological HSPCs in MF as a disease-modifying strategy [62].

CD123 (IL-3Rα) is emerging as one of the more translationally relevant targets in MF, given its expression across myeloid malignant compartments and immune–inflammatory cellular networks. The most direct clinical evidence currently comes from a phase 1/2 study of tagraxofusp (CD123-directed cytotoxin) in MF, which showed manageable tolerability without cumulative marrow toxicity and demonstrated modest spleen and symptom improvements, thereby supporting the feasibility of CD123-directed therapy in this setting [33,63]. Specifically, among 18 patients with baseline splenomegaly, two relapsed/refractory patients achieved a spleen volume reduction of at least 35%; in relapsed/refractory patients, 40% achieved a total symptom score reduction of at least 50%, with a median overall survival of 19.3 months, whereas in treatment-naive patients, 40% achieved Total Symptom Score 50 (TSS50) with a median overall survival of 26.6 months. Nevertheless, CD123-directed CAR-T or other sustained T-cell-redirected strategies remain limited by on-target/off-tumor concerns, because CD123 is also expressed on normal hematopoietic progenitors and activated immune subsets [64].

Furthermore, CAR constructs targeting the MPL (thrombopoietin receptor, TPO-R) signaling axis are currently at a proof-of-concept stage. TPO-based CAR-T cells, utilizing thrombopoietin (TPO) as the binding domain, have demonstrated efficient elimination of MPL^+^ leukemic cells in both in vitro and in vivo models, but also induce substantial cytotoxicity against normal MPL^+^ HSPCs [65,66]. This lack of selectivity suggests that MPL-directed CAR-T strategies may be more appropriately positioned as controlled myeloablative approaches or as bridging therapies prior to hematopoietic stem cell transplantation (HSCT) [67].

### 4.2. Targeting Fibrosis-Associated Cells

Beyond direct targeting of malignant clones, immunotherapeutic strategies directed at fibrosis-associated stromal cells represent an important extension in MF treatment [68]. Recent single-cell and spatial transcriptomic analyses have identified activated fibroblast populations within the PMF bone marrow microenvironment, characterized by upregulation of genes involved in collagen synthesis, extracellular matrix remodeling, and TGF-β signaling. These stromal cells not only contribute to collagen deposition but also regulate HSPC homing and immunosuppressive signaling through secretion of CXCL12, TGF-β, and other mediators [43].

Fibroblast activation protein (FAP), a canonical surface marker of activated fibroblasts, has emerged as a key target for microenvironmental intervention. FAP is highly expressed in CAFs across multiple solid tumors and fibrotic lesions, while exhibiting limited expression in quiescent normal tissues [69]. Targeting FAP—via monoclonal antibodies, bispecific constructs, or CAR-T cells—can deplete activated fibroblasts, reduce matrix stiffness, and attenuate immunosuppressive signaling, thereby facilitating microenvironmental remodeling. Preclinical studies of FAP-directed CAR-T cells have demonstrated synergistic antifibrotic and antitumor effects; however, safety concerns such as stromal disruption and cachexia remain a challenge [70,71,72].

Current evidence indicates that FAP-targeted strategies can be broadly categorized into three approaches: monoclonal antibodies or antibody-based conjugates, bispecific molecules, and CAR-T-cell therapies. First, in the context of antibody-based approaches, the early humanized anti-FAP monoclonal antibody sibrotuzumab was evaluated in a clinical study of metastatic colorectal cancer. Among 17 evaluable patients, repeated weekly administration for at least eight infusions did not result in complete or partial responses. In 24 patients who received two or more infusions, anti-sibrotuzumab antibodies were detected in three patients (12.5%) after 4–12 infusions, indicating an overall favorable tolerability profile but limited antitumor activity as monotherapy [73,74]. Subsequently, antibody platforms have evolved toward conjugate-based strategies. For instance, FAP-targeted antibody–radionuclide conjugates have demonstrated high tumor accumulation and significant antitumor efficacy in preclinical models. Second, for bispecific molecules, FAP is increasingly exploited as a spatial anchoring target to confine immune activation within FAP-positive stromal regions. FAP-4-1BBL has been shown in preclinical studies to enhance T-cell activation, proliferation, and effector function, including increased secretion of IFN-γ, IL-2, and granzyme B, and to augment tumor cell killing when combined with T-cell engagers. In a first-in-humans study (RO7122290) in patients with advanced solid tumors, increased intratumoral infiltration of CD8^+^ and Ki67^+^CD8^+^ T cells was observed, along with upregulation of T-cell activation-related genes and signatures. Among 115 treated patients, 11 achieved complete or partial responses, and the maximum tolerated dose was not reached. However, dose-limiting toxicities were reported, including grade 3 cytokine release syndrome (CRS), febrile neutropenia, and pneumonia [75]. Finally, in the context of FAP-targeted cellular therapies, strategies such as FAP-CD40 demonstrate potent, FAP-dependent CD40 activation in vitro and markedly enhance T-cell-mediated inflammatory responses in vivo, resulting in suppression of tumor growth in models such as KPC-4662-huCEA. These findings suggest that targeting FAP-positive stromal cells can both disrupt the tumor-supportive stroma and enhance host antitumor immunity. However, safety concerns remain significant [76]. Previous studies have shown that FAP-reactive T cells can recognize FAP-positive multipotent stromal cells in the bone marrow, leading to severe bone toxicity and cachexia, highlighting a clear on-target/off-tumor risk.

Clinically, FAP-CAR-T therapy has been explored in a phase I study in malignant pleural mesothelioma using intrapleural administration. Four patients were enrolled, one of whom did not receive CAR-T-cell infusion due to insufficient expansion following retroviral transduction. All treated patients underwent intensive monitoring during the first 48 h after FAP-CAR-T infusion. Despite local delivery, FAP-CAR-T cells were detectable in the systemic circulation, indicating systemic distribution. Moreover, all CAR-T cell products exhibited high specificity in vitro, which was reflected clinically by elevated levels of proinflammatory cytokines in patient sera, suggesting the presence of ongoing immune activation in vivo [77,78].

### 4.3. Targeting the Immunosuppressive Microenvironment

The CXCR4–CXCL12 axis, also known as the stromal cell-derived factor-1 axis, plays an important role in hematopoietic cell trafficking and bone marrow niche interactions. In AML, CXCR4 upregulation has been linked to stromal protection and chemoresistance, providing a rationale for CXCR4-directed strategies. However, its role in MF should be interpreted with caution. Multiple studies have shown that CXCR4 expression is downregulated on CD34-positive hematopoietic stem/progenitor cells from patients with MF, and this reduction has been associated with prognostic implications [79,80]. Therefore, CXCR4 should not be considered a straightforward clone-directed CAR-T target in MF. Instead, the CXCR4–CXCL12 axis may represent a niche-regulatory pathway whose therapeutic relevance should be evaluated through antigen-expression profiling, cellular compartment analysis, and preclinical validation [81].

The TGF-β pathway represents a central node linking immunosuppression and fibrosis. TGF-β suppresses effector T-cell proliferation, promotes Treg differentiation, and drives fibroblast activation and extracellular matrix deposition. CAR-T cells engineered to express dominant-negative TGF-β receptors or secrete TGF-β antagonists can overcome TGF-β-mediated immunosuppression, thereby enhancing persistence and effector function. This approach has been validated in multiple tumor models characterized by fibrosis and immune suppression, including pancreatic ductal adenocarcinoma, breast cancer, and hepatocellular carcinoma, providing a strong rationale for its application in MF [57,82,83,84].

MDSC- and TAM-related antigens are emerging candidates for microenvironment remodeling in MF. Single-cell and immune-phenotyping studies suggest expansion of immunosuppressive myeloid programs in PMF/MF, with enrichment of inflammatory/fibrotic and dysfunction-associated myeloid signatures. In particular, circulating and splenic MDSCs are increased in PMF and correlate with disease progression [49]. In a translational study from Moffitt Cancer Center, the CD33/CD3 bispecific T-cell engager AMV564 selectively depleted CD33^high^ MDSCs in MDS and melanoma specimens and restored T-cell activation/function, supporting the CD33 axis as a promising route for MDSC-directed intervention [44,85,86]. However, CD33/CD3 bispecific CAR-T cells and CD33 CAR-T cells that have entered human studies remain primarily focused on AML/MDS, whereas clinical strategies explicitly designed to eliminate MDSCs or TAMs are still limited.

To provide a structured overview of the therapeutic targets discussed above, we summarized the candidate targets and pathways for CAR-T-cell-based strategies in MF in Table 1. These targets are categorized according to their predominant biological roles, including clone-directed antigens, fibrosis-associated stromal targets, and immune microenvironmental or niche-regulatory pathways. This classification emphasizes that effective CAR-T-cell therapy in MF may require a multidimensional strategy that combines malignant clone elimination with remodeling of the fibrotic and immunosuppressive bone marrow niche. Importantly, given the reported downregulation of CXCR4 on MF hematopoietic stem/progenitor cells, the CXCR4–CXCL12 axis should be interpreted primarily as a niche-regulatory pathway rather than a straightforward clone-directed CAR-T target.

**Figure 3:**
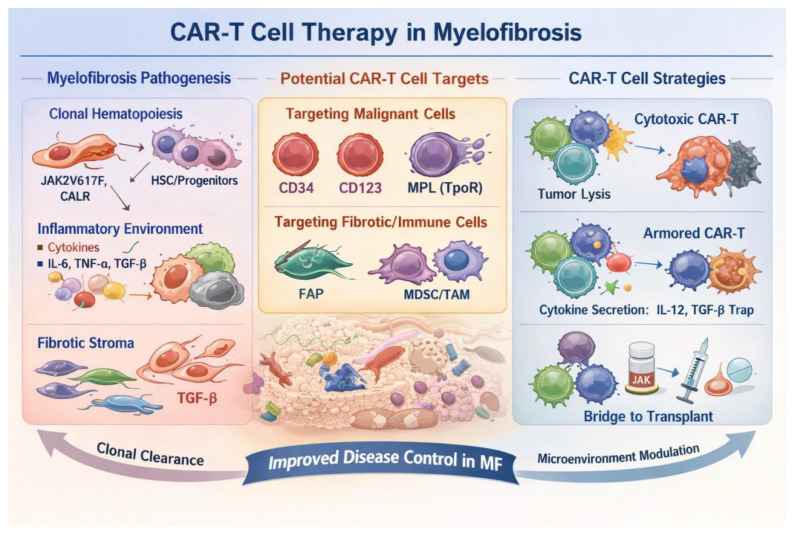
Integrated framework of CAR-T-cell therapy in myelofibrosis.

In summary, targeting the CXCR4 axis, inhibiting TGF-β signaling, and selectively depleting MDSCs/TAMs represent three complementary strategies for remodeling the immunosuppressive microenvironment in MF. Future directions will likely focus on integrating these approaches with clone-specific CAR-T therapies or enhancing microenvironmental modulation through engineered armored CAR-T cells, thereby achieving more durable immune control and disease modification. Collectively, these findings support a multidimensional therapeutic framework in MF that integrates disease pathogenesis, target selection, and CAR-T engineering strategies. A schematic overview linking these components is presented in Figure 3.

## 5. Challenges of CAR-T Therapy in Myelofibrosis

Despite the therapeutic opportunities summarized above, the clinical translation of CAR-T-cell therapy in MF remains challenging because of target overlap, fibrotic niche barriers, baseline inflammatory status, and disease heterogeneity.

The translation of CAR-T therapy in MF faces multiple challenges. First, target safety represents a major limitation, as most candidate antigens are shared with normal HSPC compartments, raising the risk of impaired hematopoietic reserve and prolonged cytopenias [87]. Second, the fibrotic bone marrow in MF is not merely a physical barrier but a pathologic niche shaped by inflammation, stromal remodeling, and immune dysregulation. Transcriptomic and single-cell analyses have demonstrated progressive enrichment of inflammatory and fibrotic gene signatures from pre-fibrotic to overt PMF, accompanied by reduced cytotoxicity scores and increased dysfunction signatures in T and NK cells. These findings suggest that infiltration in vivo and functional persistence of CAR-T cells may be constrained by both the fibrotic stromal niche and the immunosuppressive signaling network [44,88]. Third, toxicity profiles require careful consideration. MF is characterized by baseline systemic inflammation, splenomegaly, anemia, and cytopenias; therefore, patients may have reduced organ and hematopoietic reserve. In this context, therapies associated with CRS and immune effector cell-associated neurotoxicity syndrome may necessitate earlier monitoring and risk-adapted management strategies [89,90]. Finally, disease heterogeneity further complicates therapeutic development. Primary and secondary MF differ substantially in clinical phenotype, mutational landscape, and allele burden. These differences may influence antigen density, clonal architecture, and immune microenvironment composition, thereby contributing to heterogeneous CAR-T responses and increasing the likelihood of antigen escape [91,92].

## 6. Future Optimization Strategies and Combination Approaches

To overcome these barriers, future CAR-T-cell strategies in MF should integrate inflammatory control, microenvironmental remodeling, controllable safety designs, and rational sequencing with allo-HSCT.

Future optimization of CAR-T therapy for MF will likely require an integrated strategy centered on anti-inflammatory control, immune microenvironment remodeling, safety-oriented engineering, and hematopoietic stem cell transplantation. JAK inhibitors already constitute the therapeutic backbone of MF, with ruxolitinib, fedratinib, pacritinib, and momelotinib used across distinct clinical scenarios defined by splenomegaly, symptom burden, anemia, and thrombocytopenia [2]. However, CAR-T combined with JAK inhibition has not yet been systematically explored in MF, and its main current rationale lies in peritherapeutic inflammatory control rather than established disease-specific efficacy. Given that MF is a chronically inflamed myeloid neoplasm driven by aberrant JAK-STAT signaling, and that CRS is likewise amplified through cytokine networks converging on JAK/STAT pathways, peri-CAR-T administration of JAK inhibitors has a clear biological rationale for mitigating excessive inflammatory toxicity. Existing evidence comes mainly from small clinical and translational studies in CAR-T-associated CRS, in which ruxolitinib rapidly controlled steroid-refractory severe CRS and reduced circulating cytokine levels. Nevertheless, the same and subsequent mechanistic studies also suggest that ruxolitinib may dampen CAR-T expansion and cytotoxic function. Accordingly, JAK inhibitors may be better positioned as adjunctive inflammatory modulators within MF-directed CAR-T strategies, although prospective studies in MF-specific populations are still required to define the true balance between safety and efficacy [93,94].

Second, armored CAR-T strategies are expected to address the challenges posed by the fibrotic and immunosuppressive bone marrow niche. These approaches aim to enhance infiltration, survival, and persistence of CAR-T cells through incorporation of cytokine modules (e.g., IL-7/IL-15 signaling) and reprogramming of suppressive myeloid components such as MDSCs, thereby maintaining effector function within hostile microenvironments. Third, controllable CAR-T designs represent an important innovation for improving safety. By incorporating exogenous small molecules or adaptor-based systems, CAR-T activation can be pharmacologically regulated, allowing precise control over activation intensity, duration, and exposure. This strategy may significantly reduce on-target, off-tumor toxicity and long-term hematopoietic suppression. Recent studies have demonstrated that switchable CAR-T systems targeting low-selectivity antigens such as CD40 can retain antitumor efficacy while markedly reducing toxicity in preclinical models, highlighting their translational potential. This is particularly relevant in MF, where antigen overlap with normal hematopoietic or stromal cells is difficult to avoid, making controllability a critical safety prerequisite [95,96,97].

Allogeneic CAR-based cellular therapies, including universal CAR-T cells and CAR-NK cells, may help overcome key limitations of autologous CAR-T-cell therapy in MF. Autologous CAR-T products require patient-specific leukapheresis and individualized manufacturing, and their quality may be affected by prior treatment, chronic inflammation, cytopenias, and T-cell dysfunction. These issues are particularly relevant in MF, which is often associated with advanced age, inflammatory activation, splenomegaly, marrow fibrosis, and impaired hematopoietic reserve. By contrast, allogeneic CAR-T and CAR-NK cells derived from healthy donors, cord blood, or induced pluripotent stem cells may provide off-the-shelf availability, improved product consistency, shorter manufacturing time, and better scalability [98,99]. Nevertheless, universal CAR-T cells require genetic engineering, such as T-cell receptor disruption and human leukocyte antigen modulation, to minimize graft-versus-host disease and host-versus-graft rejection [18]. In contrast, CAR-NK cells may offer a more favorable safety profile, with lower risks of severe cytokine release syndrome, immune effector cell-associated neurotoxicity syndrome, and graft-versus-host disease, but may be limited by weaker expansion, shorter persistence, and potentially insufficient homing to the fibrotic marrow niche. To improve standardization and reduce interpatient variability, future strategies should include defined cell sources, automated closed-system manufacturing, standardized release criteria, validated potency assays, cryopreserved products, and biomarker-guided patient selection [37,95]. In MF, standardized assessment of antigen density, fibrosis grade, inflammatory cytokine profiles, immune-cell composition, and niche-related markers may help optimize target selection, dosing, and combination strategies.

Relatedly, starting-cell quality remains a critical determinant of autologous CAR-T-cell manufacturing success and clinical efficacy. In heavily pretreated patients, prior therapy, chronic inflammation, cytopenias, and T-cell exhaustion may compromise T-cell fitness and reduce the quality of the final CAR-T-cell product, a concern that is particularly relevant in MF because of systemic inflammation, splenomegaly, marrow fibrosis, and impaired hematopoietic reserve [100]. Potential strategies include earlier referral and leukapheresis, bridging regimens that preserve T-cell fitness, enrichment of less differentiated T-cell subsets, optimized and shortened manufacturing protocols, automated closed-system production, standardized release criteria, validated potency assays, and cryopreserved cellular products [101,102]. Off-the-shelf allogeneic CAR-T or CAR-NK platforms may further shorten treatment timelines and reduce patient-specific variability, although their safety and persistence require continued evaluation [98].

From a therapeutic perspective, CAR-T therapy in MF may be more realistically positioned as a bridging strategy to allo-HSCT rather than a definitive curative modality. Allo-HSCT remains the only potentially curative treatment for MF; however, its application is limited by high early non-relapse mortality, stringent fitness requirements, and adverse peri-transplant factors such as systemic inflammation, splenomegaly, and cachexia. Clinical practice often requires pre-transplant optimization using JAKi to ameliorate spleen size and reduce symptom burden. Multicenter studies have suggested that upfront HSCT may be associated with higher early mortality and does not consistently confer superior long-term survival compared with initial JAKi-based strategies. These findings support a stepwise approach in which systemic status is optimized prior to transplantation. In this context, if CAR-T therapy can effectively reduce inflammatory burden, ameliorate splenomegaly, and decrease malignant clone burden and profibrotic signaling in the short term, it may serve as a valuable bridging modality to improve transplant eligibility and outcomes [103,104,105].

## 7. Conclusions

To date, CAR-T-cell therapy has not yet been clinically validated in MF [1]. However, advances in understanding the immunopathology and fibrotic mechanisms of MF—particularly through single-cell and spatial transcriptomic analyses—have revealed that disease progression is characterized by amplified inflammation, enhanced immunosuppression, and progressive stromal remodeling. These findings suggest that single-modality approaches targeting either inflammation or clonal proliferation alone are insufficient for comprehensive disease control [43,44,68]. In this context, CAR-T therapy offers a novel conceptual framework by integrating targeted cytotoxicity with microenvironmental modulation through cellular engineering. Nevertheless, current research in MF remains largely at the exploratory and proof-of-concept stage.

Future breakthroughs will likely depend on the integration of precision target selection and microenvironment-directed strategies. On one hand, antigen selection must be optimized based on molecular stratification (e.g., JAK2, CALR, MPL driver mutations and high-risk mutational profiles) to enhance tumor specificity and safety [87]. On the other hand, modulation of key microenvironmental pathways—including the CXCR4–CXCL12 axis, TGF-β signaling, and myeloid suppressive networks—should be incorporated into CAR-T design through armored constructs or combination therapies to improve functional persistence within the fibrotic bone marrow niche [57].

In summary, CAR-T therapy represents a potential paradigm shift in MF, moving from symptomatic management toward potentially curative strategies. However, successful clinical translation will require coordinated optimization of antigen specificity and dual targeting of immune and stromal components. With continued advances in cellular engineering and risk-adapted therapeutic strategies, CAR-T therapy holds promise for delivering clinical benefit in selected high-risk or refractory MF populations.

## Figures and Tables

**Table 1:** Potential targets and therapeutic strategies for CAR-T-cell therapy in myelofibrosis.

Category	Candidate Target	Therapeutic Rationale	Main Limitation
Malignant clone-directed targets	CD34, CD123, MPL	Direct elimination of disease-driving hematopoietic clones	Antigen overlap with normal HSPCs and risk of hematologic toxicity
Fibrosis-associated stromal targets	FAP, TGF-β-related pathways	Remodeling of fibrotic bone marrow niche and reduction in stromal support	Stromal toxicity and broad biological functions
Immunosuppressive microenvironment targets	CXCR4–CXCL12, MDSC/TAM-related targets, CD33	Disruption of protective niche signals and reversal of immune suppression	Limited MF-specific evidence and risk of off-tumor toxicity
Immune-function regulatory pathways	PD-1, TIM-3, LAG-3, TIGIT	Restoration of exhausted T-cell function and enhancement of CAR-T persistence	More suitable as combination strategy than direct CAR-T target

## Data Availability

No new data were created or analyzed in this study. Data sharing is not applicable to this article.

## Abbreviations

allo-HCT	Allogeneic Hematopoietic Stem Cell Transplantation
AML	Acute Myeloid Leukemia
BCMA	B-Cell Maturation Antigen
BiTE	Bispecific T-Cell Engager
BMF	Bone Marrow Fibrosis
CAF	Cancer-Associated Fibroblast
CAR-T	Chimeric Antigen Receptor T Cell
CD123	Cluster of Differentiation 123
CR	Complete Remission
CRS	Cytokine Release Syndrome
CXCL12	C-X-C Motif Chemokine Ligand 12
CXCR4	C-X-C Motif Chemokine Receptor 4
ECOG	Eastern Cooperative Oncology Group Performance Status
ET	Essential Thrombocythemia
FAP	Fibroblast Activation Protein
HSCT	Hematopoietic Stem Cell Transplantation
HSPC	Hematopoietic Stem and Progenitor Cell
ICANS	Immune Effector Cell-Associated Neurotoxicity Syndrome
IFN-γ	Interferon Gamma
IL-1β	Interleukin 1 Beta
IL-2	Interleukin 2
IL-6	Interleukin 6
JAK-STAT	Janus Kinase–Signal Transducer and Activator of Transcription
JAKi	Janus Kinase Inhibitor
LAG-3	Lymphocyte Activation Gene 3
LSC	Leukemic Stem Cell
MDS	Myelodysplastic Syndrome
MDSC	Myeloid-Derived Suppressor Cell
MF	Myelofibrosis
MPL	Myeloproliferative Leukemia Protein
MPN	Myeloproliferative Neoplasm
NF-κB	Nuclear Factor Kappa B
NK	Natural Killer
ORR	Overall Response Rate
OS	Overall Survival
PD-1	Programmed Cell Death Protein 1
PDGF	Platelet-Derived Growth Factor
PFS	Progression-Free Survival
PI3K–AKT–mTOR	Phosphoinositide 3-Kinase–AKT–Mammalian Target of Rapamycin
PMF	Primary Myelofibrosis
PMN-MDSC	Polymorphonuclear Myeloid-Derived Suppressor Cell
PV	Polycythemia Vera
scFv	Single-Chain Variable Fragment
SMF	Secondary Myelofibrosis
STAT3	Signal Transducer and Activator of Transcription 3
TAM	Tumor-Associated Macrophage
TGF-β	Transforming Growth Factor Beta
TIGIT	T-Cell Immunoreceptor with Ig and ITIM Domains
TIM-3	T-Cell Immunoglobulin and Mucin Domain Containing 3
TNF-α	Tumor Necrosis Factor Alpha
TPO	Thrombopoietin
Treg	Regulatory T Cell
TRAF–NF-κB	Tumor necrosis factor receptor-associated factor–nuclear factor kappa B
TSS50	50% Reduction in Total Symptom Score
